# Snip  & Stitch: a simple and accessible correction for the pupil foreshortening error

**DOI:** 10.3758/s13428-026-02980-8

**Published:** 2026-03-30

**Authors:** Koert H. Stribos, Damian Koevoet, Yuqing Cai, Marnix Naber, Christoph Strauch

**Affiliations:** Experimental Psychology, Helmholtz Institute, Utrecht University, Utrecht, The Netherlands

**Keywords:** Pupil size, Pupil foreshortening error, Gaze, Saccade

## Abstract

Modern eye trackers allow easy measurement of pupil size, a powerful physiological marker of cognitive processing. A major hindrance to using pupillometry is the so-called pupil foreshortening error (PFE). The PFE is the apparent change in pupil size that results from a change in the angle of the eye relative to the camera, despite the pupil remaining constant in reality. To prevent this, experimenters often rely on fixed gaze, limiting design flexibility. Existing correction methods necessitate specific geometric properties of the setup, often unavailable for existing datasets, and do not work for all tracker types. We here introduce ‘Snip  & Stitch’, a straightforward, accessible, and easy-to-implement correction for the PFE. Specifically, Snip  & Stitch corrects the PFE by ‘snipping’ pupil size changes during a saccade, and ‘stitching’ pupil size back by subtracting the difference between pre- and post-saccadic pupil size. Our results demonstrate that this simple method reduced the PFE by an estimated 71–81%. Snip  & Stitch is openly available online. We recommend Snip  & Stitch, especially for experiments in which participants make one saccade before pupil size is compared, and argue that the method can be easily applied to experiments in which participants make up to five saccades in sequence. Limitations and possible further improvements are discussed. Together, Snip  & Stitch allows researchers to employ pupil size as an outcome measure in a wide range of tasks and setups.

**Supplementary Information:**

The online version contains supplementary material available at 10.3758/s13428-026-02980-8.

## Introduction

Pupillometry, the measurement of pupil size changes over time, is a powerful physiological marker of perception and cognition (reviewed in Strauch et al., 2022; Mathôt, 2018; Mathôt and Van der Stigchel, 2015; Beatty and Lucero-Wagoner, 2000; Bumke, 1911; Laeng and Alnaes, 2019; Koevoet et al., 2024a). As such, it can finely track the allocation of covert attention (e.g., Naber et al., 2013; Mathôt et al., 2013) and visual sensitivities (Cai et al., 2025). Furthermore, due to its tight coupling with mental effort (Bumke, 1911; Hess and Polt, 1964; Kahneman and Beatty, 1966), pupil size provides insights into a wide range of cognitive processes, including attention (Strauch et al., 2022), working memory (Koevoet et al., 2024a), affective processes (Partala & Surakka, 2003), and decision-making (Murphy et al., 2014; Strauch et al., 2020). While early pioneers relied on rulers, photographs, or magnifying glasses and telescopes (Berrien & Huntington, 1943; Bumke, 1911; Strauch, 2024), current-day researchers can make use of video-based eye trackers reporting pupil size as well as gaze position automatically at frequencies of up to 2000 Hz (Carter & Luke, 2020).

**Fig. 1:**
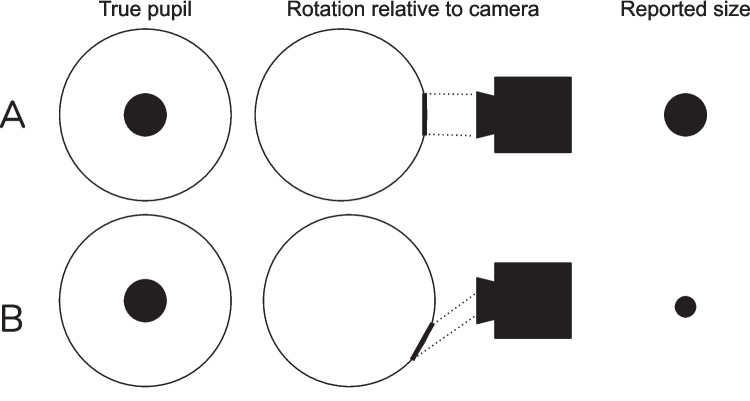
Schematic display of the pupil foreshortening error (PFE). At equal physiological pupil size (column 1), a static video-based eye tracker picks up a pupil that is obscured to different extents due to its rotation in relation to the camera (column 2; **A** no obscuration, **B** partly obscured). This causes the eye tracker to report pupil size to be lower in B than in A, even though physiological size is identical

When designing pupillometry experiments, multiple potential confounding factors ranging from experimental design to statistical analysis should be considered (Fink et al., 2023; Mathôt and Vilotijević, 2023; Reilly et al., 2024). Most prominently, changes in illumination let the pupil change (up to about 4 mm) at magnitudes that substantially exceed changes related to mental effort (up to about 0.5 mm), to give an example (Beatty, 1982; Beatty & Lucero-Wagoner, 2000; Laeng et al., 2012). Such low-level sensory confounds can be prevented by making stimuli iso- or equiluminant (e.g., Koevoet et al., 2023; Koevoet et al., 2024b), or may be addressed post-hoc by removing effects of luminance on pupil size using modeling approaches (Cai et al., 2023).

Another, perhaps lesser-known, potential confound when investigating cognitive effects on pupil size is the pupil foreshortening error (PFE). The PFE entails apparent changes in pupil size that do not result from actual changes in pupil size, but instead from changes in the angle of the eye relative to the camera (Fig. 1). Specifically, when gaze is shifted, the observed eye rotates in relation to the eye tracker camera. This causes the projected image of the pupil to become smaller and partially obscured, changing the estimate of pupil diameter reported by the eye tracker by up to 1 mm - but note that this depends on gaze location and eye tracker (Brisson et al., 2013; Gagl et al., 2011; Hayes & Petrov, 2016). Additionally, the effect of gaze angle rotation on the pupil size reported by the eye tracker is further modulated when the image falling from the eye onto the eye tracker is refracted by transparent surfaces, such as the cornea, a participant’s glasses, or a mirror used in the stimulus presentation setup (Hayes & Petrov, 2016; Petersch & Dierkes, 2022). As a consequence of the substantial PFE effect, pupil size changes can usually only be meaningfully interpreted if gaze position is equal across experimental conditions. This is why guidelines generally argue for constant gaze position in pupillometric experiments or for keeping gaze location equal across conditions (Mathôt and Vilotijević, 2023). This, however, heavily limits the types of tasks that can be studied with pupillometry (Hayes and Petrov, 2016).

When controlling the PFE experimentally is not feasible, or simply was not thought of at the time of collection, an alternative to experimental control is to correct the PFE (mapping it) or to make PFE-free measurements from the get-go (e.g., rotation-aware eyeball models). We describe extant methods in the following paragraph.

Firstly, the PFE can be systematically mapped out across all gaze positions. This mapping can be derived using geometry, or by data-driven calibration (Gagl et al., 2011; Hayes & Petrov, 2016). Once the effect of gaze position on pupil size is known, the PFE can be corrected for. Nevertheless, such a correction necessitates researchers to calibrate their setup. For the case of a geometrically derived PFE map, calibration is done by measuring all spatial properties of that setup, including distances between the participant, camera, and screen (Hayes & Petrov, 2016), for the case of data-driven calibration, calibration is performed by letting participants move their gaze across the screen and recording the resulting pupil size change. Using such methods to correct the PFE still poses limits to experimental setups. For instance, as recording the eye through refractive and reflective surfaces adds complexity to the PFE surface, current geometric models cannot be generalized to eye tracking setups where refraction or reflection is present, such as the popular tower mount EyeLink 1000 (Sr Research, 2010) or eye tracking setups designed for use in dichoptic viewing conditions (e.g. Brascamp and Naber, 2017) or during fMRI scanning (Kanowski et al., 2007). Moreover, differences between participants, such as glasses worn to correct vision, also introduce refraction, leading to the recommendation to exclude participants who wear glasses when using a geometrically derived PFE map (Hayes & Petrov, 2016). Further compounding this issue, differences in eyeball shape lead to disparities among participants in the PFE map. This gets even more complicated if the setup geometry changes, e.g., when moving participants and no chinrest can be used. Performing a per-participant calibration, where participants spend a few minutes moving their gaze over the to-be-corrected surface (Gagl et al., 2011), should take care of inter-participant differences in the PFE surface. However, saccades themselves have been associated with sizable and consistent pupil size changes, independent of the PFE (e.g. Jainta et al., 2011; Koevoet et al., 2024b; Knapen et al., 2016), any such calibration therefore ’corrects’ parts of the pupillary signal that are inherently not caused by the PFE but instead represent ’real’ pupillary change (for a similar point see Mathôt et al., 2015a). Thus, such approaches may cause pupillometrists to inadvertently ’correct’ actual (cognitive) effects away.

Alternatively (and ideally), one makes measurements that are not affected by the PFE. Extant work attempts this by constructing a 3D model of the eye from camera input (e.g.,Dierkes et al., 2018), which demonstrably reduces the PFE (Petersch & Dierkes, 2022). In theory, such a 3D eye model can be extended to include reflection and refraction from any surface to also correct the PFE in eye tracking setups where such factors are present. However, 3D eye models cannot generalize between participants due to some wearing glasses, or differences in their cornea’s refractive properties (Guestrin & Eizenman, 2006). Indeed, per-participant calibration is required to make PFE-free measurements. Other correction methods attempt PFE-free measurement using proprietary solutions, such as a deep neural network (Pfeffer & Dierkes, 2024). Proprietary solutions require minimal effort from their users to be applied but come at the cost of users not knowing what is going on underneath the hood, which may also lead to inadvertent ’corrections’ of actual cognitive effects. Crucially, proper validation of such a solution is impossible, due to its proprietary nature.

Together, existing methods of correcting the PFE are powerful, yet usually complex, cumbersome, not always applicable, and not easy to implement (in many contexts).

We here introduce an easy-to-use and straightforward alternative to existing PFE corrections: Snip & Stitch. We capitalize on the fact that, in most experiments, gaze is primarily displaced by very fast and ballistic eye movements, called saccades (Findlay & Gilchrist, 2003). In contrast, pupil size changes are relatively sluggish (Strauch et al., 2022), which should allow for dissociating saccade-induced apparent changes (i.e., PFE) from actual pupil size changes in the obtained signal. This allowed us to correct for the PFE by using pupil sizes directly preceding and following saccades.

Snip & Stitch is remarkably simple (Fig. 3):

1. Pupil size changes that were assumed to be caused by gaze shifts (i.e., between saccade on- and offset) are ’snipped’ and discarded.
2. Pupil size after saccade offset is ’stitched’ to match pupil size before saccade onset, and the introduced short cut-out intervals in the pupil time series for saccades can be patched using interpolation, just like the common standard for blink correction (Mathôt and Vilotijević, 2023).

**Fig. 2:**
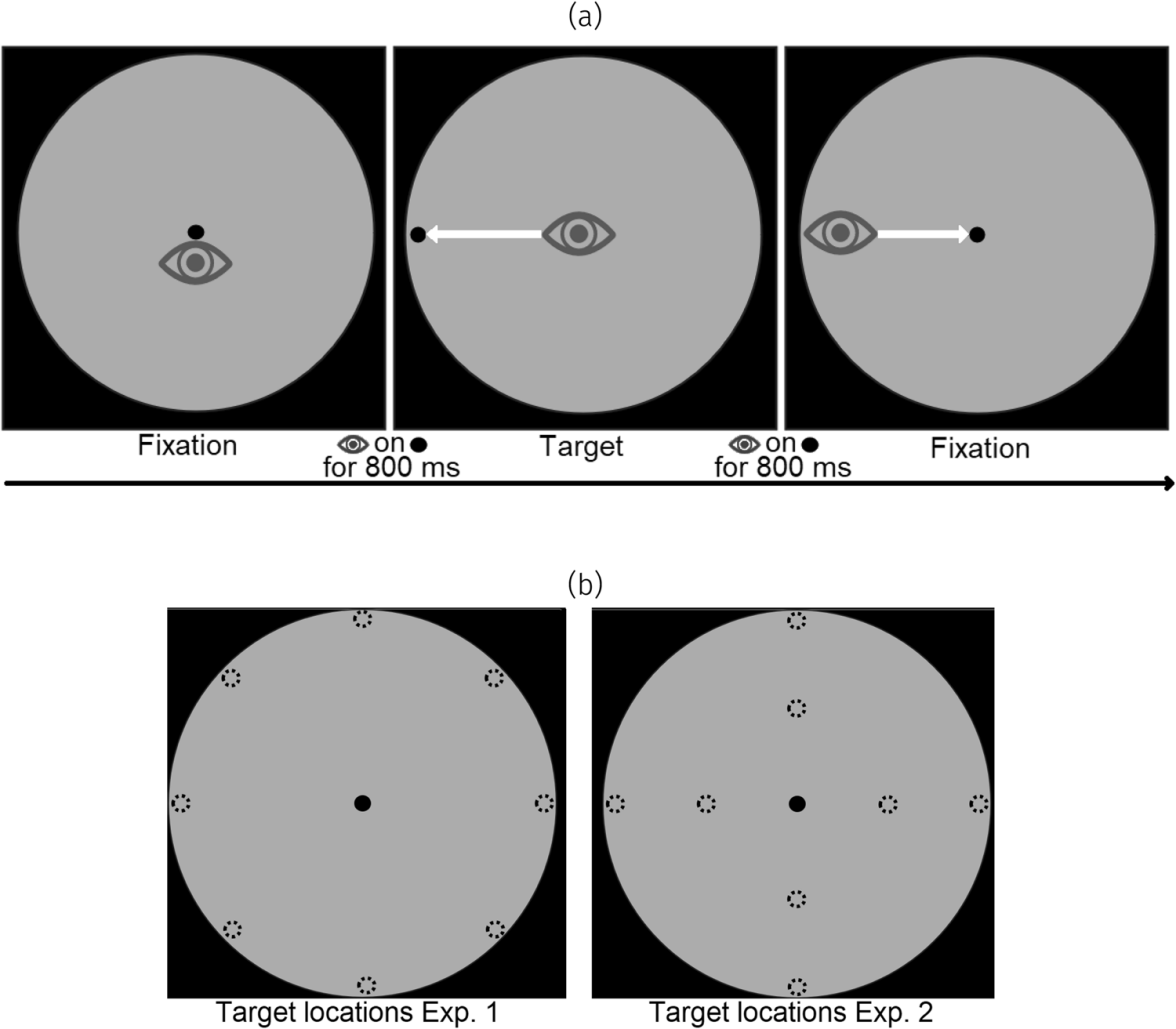
**(a)**: Overview of a typical trial in Experiment 1 and 2. Each trial started with a central target. After 800 ms of gaze on the central target, a peripheral target was presented, which the participant subsequently saccaded to. After 800 ms of looking at the peripheral target, the target was replaced with the central target, and participants saccaded back thereto. **(b)**: overview of all peripheral target locations used in Experiment 1 (*left*) and 2 (*right*). For Experiment 1, the *gray circle* had a radius of 10.5$^\circ$, and targets appeared at 10$^\circ$. For Experiment 2, the *gray circle* had a radius of 20.5$^\circ$, and targets appeared at 10$^\circ$ or 20$^\circ$

Two experiments were conducted to estimate the PFE and to test Snip  & Stitch using two different state-of-the-art eye trackers. Participants herein saccaded from a central target towards a peripheral target, and then back to the central target (Fig. 2). To preface results, Snip  & Stitch reduced measurement error after one saccade by an estimated 71–81%. The simple nature of our correction makes it applicable to a wide range of eye trackers, participants, and experiments without needing extensive, cumbersome and error-prone calibration.

## Dataset

### Participants

Five participants with normal or corrected-to-normal vision (*M*$_{age}$ = 27.4, range: [24–33]; one female, four males) took part in Experiments 1 and 2. All participants were students or employees of the division of Experimental Psychology at Utrecht University, and three are authors of this paper (K.H.S., D.K., C.S.). The experimental procedure was approved by the ethical review board of Utrecht University’s Faculty of Social Sciences (approval code: 24-0319).

### Apparatus and eye tracker

For Experiment 1, gaze position and pupil size of both eyes were measured with a video-based desktop mount EyeLink 1000 Plus tracker (SR Research, Mississauga, Ontario, Canada), recording at 2000 Hz. Only data from the left eye were used for analyses. Stimuli were presented on a Asus ROG PG278Q (2560 x 1440, 100 Hz) monitor using PyGaze (Dalmaijer et al., 2014) with PsychoPy (v2021.2.3) (Peirce, 2007) as back-end. Participants were seated in a chin-and-headrest 68 and 75 cm from the monitor in Experiments 1 and 2, respectively. A nine-point calibration and validation were conducted at the start of the experiment and between blocks whenever the participant or researcher felt the task behaved unresponsive.

For Experiment 2, gaze position and pupil size were tracked monocularly for either the left or right eye using an EyeLink 1000 Plus Tower Mount (SR Research, Mississauga, Ontario, Canada) tracker at 500 Hz. Stimuli were presented using PyGaze (Dalmaijer et al., 2014) again with PsychoPy (Peirce, 2007) as back-end on a OLED LG TV (1,920 $\times$ 1080; 60 Hz). A five-point calibration and validation were conducted at the start of the experiment and between blocks whenever a participant or researcher felt the task was unresponsive.

### Stimuli and procedure

In Experiments 1 and 2, participants completed a saccade task. Participants were instructed to saccade to a peripheral target from the center, and shortly thereafter to saccade back to the central target.

In Experiment 1, stimuli consisted of a black central target (0.6 $^\circ$ radius) presented on the center of the screen, followed by one of eight equally spaced black targets (also 0.6$^\circ$ radius) at an eccentricity of 10$^\circ$ from the central target. Targets were presented in a dim gray circle (RGB: 30;30;30) with a radius of 10.5$^\circ$. This ensured that the local luminance at each peripheral target was equal. This circle, with other stimuli on top, was centrally presented on the monitor on a black background, corresponding to the black wall of the lab surrounding the monitor. Each trial began with the central target, which was subsequently offset and replaced by one of the eight peripheral targets. Participants were instructed to saccade towards the peripheral target as fast as possible. A target was considered successfully fixated if gaze position was consecutively detected for 800 ms within 3$^\circ$ of the target. Then, the peripheral target was removed and replaced with the central target, prompting participants to saccade back to the center and begin a new trial. Each participant completed a total of 400 trials, split into four blocks. Recalibration was performed whenever the participant’s gaze did not trigger the next (phase of a) trial as intended.

**Fig. 3:**
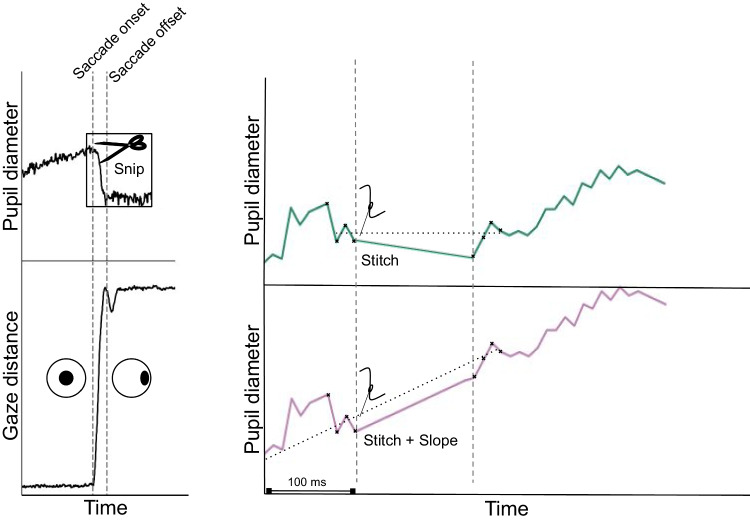
Overview of two variations of Snip  & Stitch. For every saccade, as detected by an external algorithm, pupil size trace is snipped, and stitched back. The simplest version of Snip  & Stitch assumes that pupil size does not change during this saccade, and stitches pupil size after the saccade so that the median pupil size of the four subsequent samples matches that of the four preceding samples here highlighted with *black crosses* (*top right*). The second method additionally estimates pupil size change during the saccade by fitting a slope to the last 100 ms before saccade onset, and stitches pupil size after the saccade at a modulated value (*bottom right*), thus additionally modeling pupil size change during the saccade. Values between saccade on- and offset are interpolated linearly between last and first valid sample

Experiment 2 was identical to Experiment 1, except that saccade targets were positioned at only cardinal (not diagonal) directions from the center at either 10 or 20$^\circ$ from the center. To accommodate targets presented at higher eccentricity, the radius of the dim gray circle was extended to 20.5$^\circ$.

### Pre-processing

All data were processed and analyzed in Python 3.9 using custom scripts, openly available at https://osf.io/nu75x. We first converted pupil size from arbitrary EyeLink units to millimeters using an artificial eye (provided by the manufacturer; similar to Wilschut and Mathôt, 2022; Hayes and Petrov, 2016), as using actual pupil diameters allows for a direct comparison of effect sizes of pupillary change across studies (Beatty & Lucero-Wagoner, 2000). For both eye trackers used across our experiments, we placed the artificial eye in front of the eye tracker, approximately at the location where a participant’s eye would be. The pupil size in arbitrary EyeLink units reported for the artificial eye was then used to create a conversion factor for that eye tracker.

Saccade detection was performed using I2MC, a noise-robust fixation detection algorithm (Hessels et al., 2017). We discarded trials longer than 3000 ms, trials with blinks during saccades (as detected by each EyeLink apparatus), and four additional trials that exhibited highly implausible pupil size changes upon visual inspection (i.e., likely due to undetected blinks). In total, 76.6% and 77.8% of trials (1,622 and 1,639) were retained for Experiments 1 and 2, respectively.

## Quantifying the pupil foreshortening error

We quantified the extent of PFE to compare uncorrected PFE to variation introduced by our Snip  & Stitch method. For this purpose, we used all trials with exactly two saccades (i.e., one forward saccade lands on the target and one return saccade lands on the center, see Fig. 2a center and right). For these trials, saccades are accompanied by a substantial abrupt change in recorded (but not actual) pupil size, which reflects PFE between center and periphery (in principle following the logic in Gagl et al., 2011) (Fig. 1). We applied Snip & Stitch + Slope (see the following section) to obtain PFE estimates for these trials (i.e., the average correction value used for that trial). Critically, we inverted the values for return saccades. This yielded a PFE estimate per trial.

As expected, we observed a clear PFE in both Experiment 1 (10$^\circ$, table mount; *Mdn* = 0.051 mm; 95% CI = [0.028–0.089] and in Experiment 2 (10$^\circ$ and 20$^\circ$, tower mount; *Mdn* = 0.032 mm and 0.080 mm, respectively; 95% CI = [0.011–0.066] and [0.018–0.158] respectively). As we used different eye trackers for Experiments 1 and 2, the PFE differed between the two setups. Specifically, we observed a larger PFE in Experiment 1 compared with Experiment 2 (Mann–Whitney *U* = 1352, *p* < .001, two-sided) with targets at the same eccentricity (10$^\circ$). In line with earlier work, this demonstrates that the type of eye tracker modulates the extent of the PFE. Moreover, we expected larger absolute PFE at higher saccade amplitudes because the angle between the eye and the eye tracker increases. In line with this, we observed a larger PFE when participants saccaded toward targets relatively far (20$^\circ$) compared with targets relatively near (10$^\circ$) to screen center (Mann–Whitney *U* = 219,314, *p* < .001, one-sided). For completeness, we report results separately for each target location in the Supplementary Material (Fig. 1). Together, we found a substantial PFE in Experiments 1 and 2, and the extent of the PFE depended on the type of tracker as well as the eccentricity of gaze position change.

## Introducing Snip  & Stitch

To correct for the PFE, we here introduce Snip  & Stitch, a simple and easy-to-implement method to correct the PFE (see Fig. 3 for a visualization). We will start with explaining the simplest version of Snip  & Stitch.

Snip  & Stitch relies heavily on accurate saccade detection, and corrects for PFE introduced by (saccadic) eye movements (here detected with the state-of-the-art I2MC algorithm (Hessels et al., 2017)). Because saccade detection is vital to our method, we wanted to ensure that poorly detected saccade on- and offsets did not cause confounds. For example, if saccade onsets are detected ’too late’, the PFE has already been introduced in the pupillary signal, causing us to misestimate the baseline PFE as well as the efficacy of our correction. To circumvent such cases, we systematically varied the time window we selected immediately prior saccadic onset, and immediately following saccadic offset. We calculated median pupil sizes within these windows. To determine the time window ranges, we used Nelder–Mead optimization (Gao and Han, 2012, using’*minimize*’fromSciPyv1.14.0), aiming to minimize the leftover error after correction in the data from Experiment 1 (see below for how this was quantified). Testing indicated that extending saccade on- and offset is best kept minimal. We opted for extending saccade on- and offsets with one sample to account for saccades starting mid-sample, and for a time window of four samples to account for between sample-variation. Evidently, Snip  & Stitch calculates pre- and post-saccadic window size by taking the median of the first through fourth samples outwards from saccade on-, and offset, and assumes pupil size difference introduced by the saccade to be PFE only, and thus used as PFE estimate.

The simplest implementation of Snip  & Stitch subtracts estimated PFE from all pupil sizes after saccade offset. Pupil size data during the saccade are subsequently linearly interpolated.

In our experiments, the central spot served as a reference point for the PFE that allowed to quantify Snip  & Stitch performance. Whenever looking at this central reference point, there is no PFE (relative to the central reference point). Participants performed saccades from this central reference point to a peripheral target and back. Upon returning to the central reference point, we compared the Snip  & Stitch-corrected pupil trace to the raw pupil trace as recorded by the eye tracker. This difference quantified the leftover error after applying Snip  & Stitch. If Snip  & Stitch removed the PFE perfectly, there would be no difference between corrected and uncorrected pupil size at the end of the trial.

For the first analyses, we used only trials where exactly two saccades were performed (i.e., one forward saccade lands on the target and one return saccade lands on the center perfectly, see Fig. 2a center and right). As the second saccade in each of these trials returned to the starting point of the first saccade, the PFE of the first saccade is opposite to the PFE of the second saccade, effectively canceling each other out. We halved the difference between corrected and uncorrected pupil size to quantify leftover error *per saccade* (as our non-halved estimate reflects the leftover error of two saccades otherwise).

**Fig. 4:**
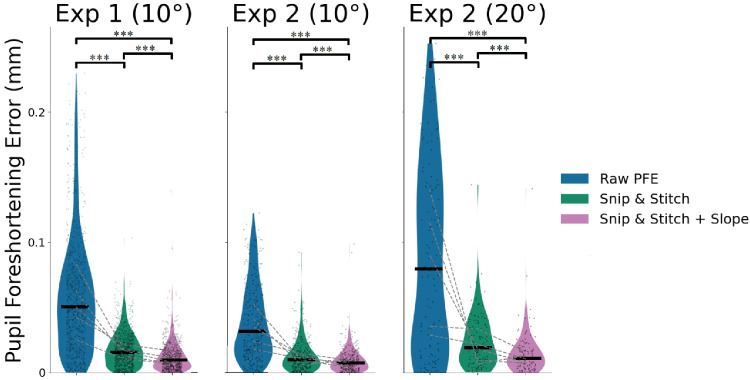
Observed PFE across targets for trials with two saccades (*blue*) in comparison to estimated leftover error on return to central target after applying (1) simple Snip & Stitch correction (*green*) and (2) Snip & Stitch correction + Slope (*purple*). *Black dots* represent individual trials. *Dashed lines* represent differences between participant medians. ***p < .001 (one-sided)

Snip  & Stitch substantially reduced the PFE across all conditions (Fig. 4). Across all trials in two experiments, we observed a 70.9% reduction of the PFE. To formally test this, we used one-sided Wilcoxon signed-rank tests to evaluate the reduction per experimental condition. Results demonstrated a significant PFE reduction for both Experiment 1 (*W*(985) = 42,370, *p* < .001) and Experiment 2 (at 10$^\circ$: *W*(349) = 6750, *p* < .001; at 20$^\circ$: *W*(118) = 819, *p* < .001). Leftover error after applying Snip  & Stitch for all trials included in the analysis is plotted in Fig. 4. Our results demonstrate that this simple implementation of Snip  & Stitch is able to correct for the PFE across different eye trackers as well as for saccades at different amplitudes.

### Snip  & Stitch + Slope: correcting for pupil size change during saccades

From the moment the eyes start to move (i.e., saccade onset) until they land at the peripheral target (i.e., saccade offset), the eyes are in-flight. The median time the eyes were in-flight (i.e., saccade duration) in trials with two saccades in Experiments 1 and 2 were 36.0 ms (*M* = 41.0, *SD* = 17.8) and 54.0 ms (*M* = 61.7, *SD* = 31.8), respectively. During this in-flight period, Snip  & Stitch, as introduced above, assumed the absence of actual pupil size change. By contrast, extant work suggests that this assumption does not hold because pupil size changes as a function of saccade preparation (Jainta et al., 2011; Koevoet et al., 2023, 2024b) and given the relative sluggishness of pupillary responses (Mathôt and Vilotijević, 2023; Strauch et al., 2022), this increase likely continues in-flight. Additionally, presaccadic attention initiates the pupillary light response to anticipate brightness at the landing location (Koevoet et al., 2025; Mathôt et al., 2015b), which also likely affects pupil size in-flight. Based on this evidence for pupillary change during saccades, we suspected that part of the leftover error that remained after Snip  & Stitch reflected actual changes in pupil size during saccades. To account for this possibility, we estimated pupil size changes during the saccade by first fitting a linear function to reported pupil sizes up to 100 ms before saccade onset. We then used this function to estimate intra-saccadic pupil size changes to better estimate pupil size at saccade offset (i.e., by multiplying the fitted slope by saccade duration). We then subtracted this value from the PFE estimate used to correct pupil size.

Snip  & Stitch + Slope improved the correction for the PFE even further. Overall, 81.1% of the total PFE was ameliorated using this extended method. We found a reduction in PFE for both Experiment 1 (*W*(985) = 23,942, *p* < .001), and Experiment 2 (at 10$^\circ$: *W*(349)=3,233, *p* < .001; at 20$^\circ$ (*W*(118) = 452, *p* < .001). Additionally, the reduction in PFE with Snip  & Stitch + Slope was significantly higher than that with the simple Snip  & Stitch (Experiment 1: *W*(985) = 78,229, *p* < .001; Experiment 2 at 10 $^\circ$: *W*(349) = 18,154, *p* < .001; Experiment 2 at 20 $^\circ$: *W*(118) = 1,368, *p* < .001). Thus, estimating the pupil’s change *during* the saccade further reduced the PFE.

### Systematic buildup across saccades

In the experiments reported above, participants were explicitly instructed to perform two saccades per trial. In this scenario, Snip  & Stitch was able to substantially reduce the PFE. Many cognitive psychology/neuroscience tasks only require a single saccade per trial, such as antisaccade (Munoz & Everling, 2004), oculomotor capture (Theeuwes et al., 1998), memory-guided saccade (Hikosaka & Wurtz, 1983), and saccade planning/preference (Koevoet et al., 2024b) tasks. However, we wanted to assess how well Snip  & Stitch performs when aiming to correct for a sequence of saccades. This is particularly relevant for potential paradigms wherein multiple saccades are executed without returning back to a point of origin (e.g., the central target in the current study). This allowed us to evaluate whether the leftover error introduced by Snip  & Stitch was systematic, i.e., pupil size becoming increasingly over-/under-estimated.

To this end, instead of analyzing a single trial at a time, we applied Snip  & Stitch to chains of up to 30 saccades. We extrapolated from our earlier findings by combining successive valid trials into longer pseudotrials. To increase chain lengths, trials with more than two saccades (e.g., corrective saccades) were now also included for analysis. As described before, a (pseudo) trial starts and ends with gaze at the central target, which means that no, or a minimal, effect of the PFE on pupil size should exist between these two points. Any leftover difference between the corrected and uncorrected pupil size at the end of a (pseudo) trial therefore represents the leftover error introduced by correcting the PFE with Snip  & Stitch + Slope across a saccade sequence.

**Fig. 5:**
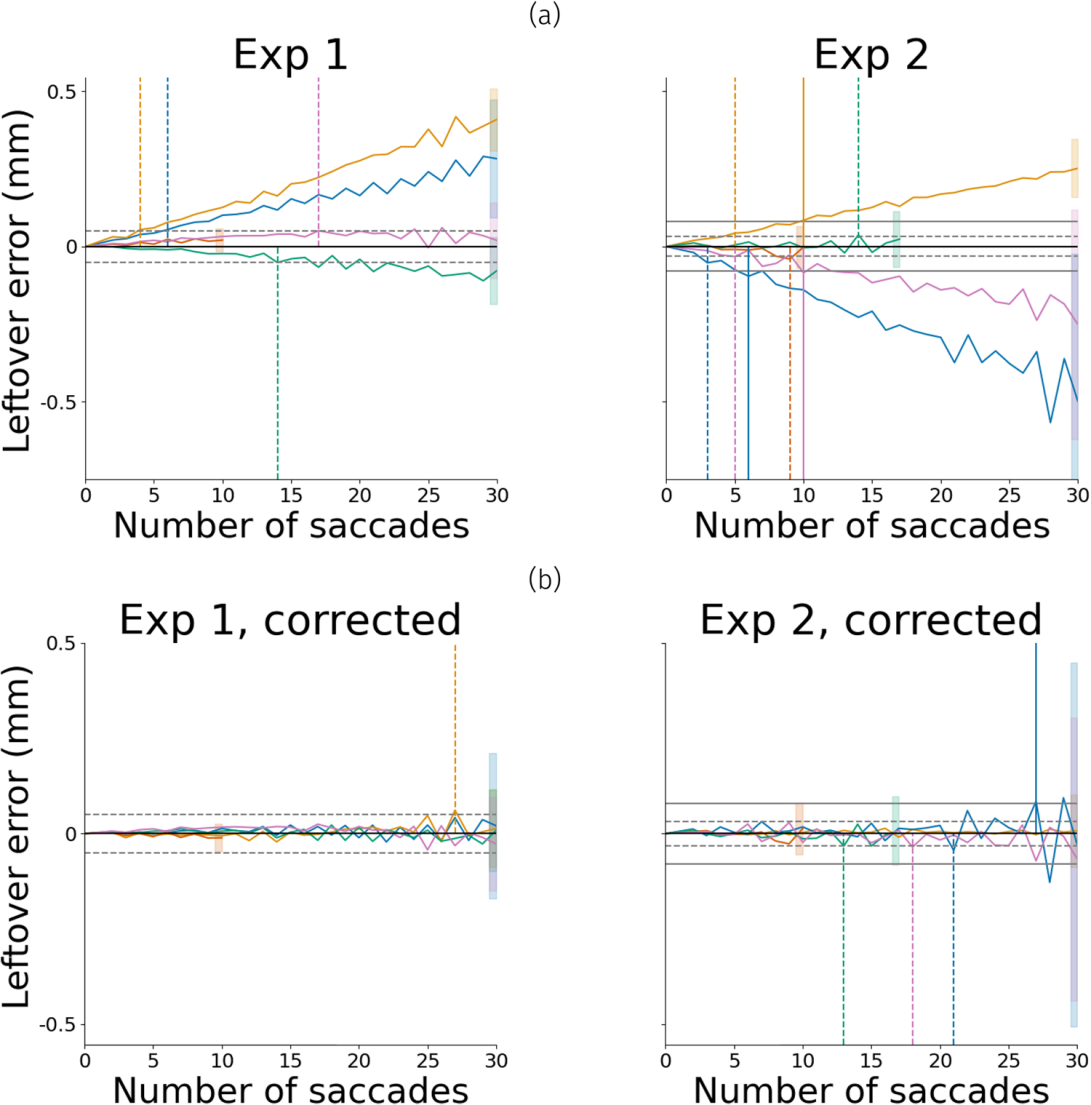
Average build-up of leftover error per participant before **(a)** and after **(b)** correction as described in the ’Correcting build-up’ section. *Horizontal gray line* (dashed 10-$^\circ$ condition, solid 20-$^\circ$ condition) is the median observed PFE across that eccentricity for the entire experiment. For each participant, a *vertical line* indicates the number of saccades where the built-up error after applying Snip  & Stitch exceeded the median observed PFE without any correction applied. Additionally, the mean ± SD is given as a *shaded bar* at the endpoint of each participant’s moving average

We chained all combinations of up to 15 successive trials to generate 9,216 and 11,619 pseudotrials for Experiments 1 and 2, respectively. Note that for Experiment 2, chains consisted of trials where saccade targets were either at 10$^\circ$ or 20$^\circ$ eccentricity. From each pseudotrial, we calculated leftover error across saccade chains to visualize the build-up. The results are plotted per participant in Fig. 5a (for a visualization of build-up per participant and direction see Supplementary Fig. 1). These data showed that the leftover error had already started to build up systematically across a few saccades.

### Correcting for the systematic build-up across saccades

Leftover error built up differently per experiment and participant, but was consistent within each participant in each experiment, exhibiting a linear trend (Fig. 5a). To correct for this build-up of leftover error across consecutive applications of Snip  & Stitch, we first fit an ordinary least-squares linear model. This model estimated the build-up leftover error after consecutive saccades across pseudotrials. This allowed us to obtain a correction factor (i.e., $\beta$ from fitting $y = \beta x$) related to each participant per experiment. We then applied this correction factor to the pseudotrials from which it was generated.

**Fig. 6:**
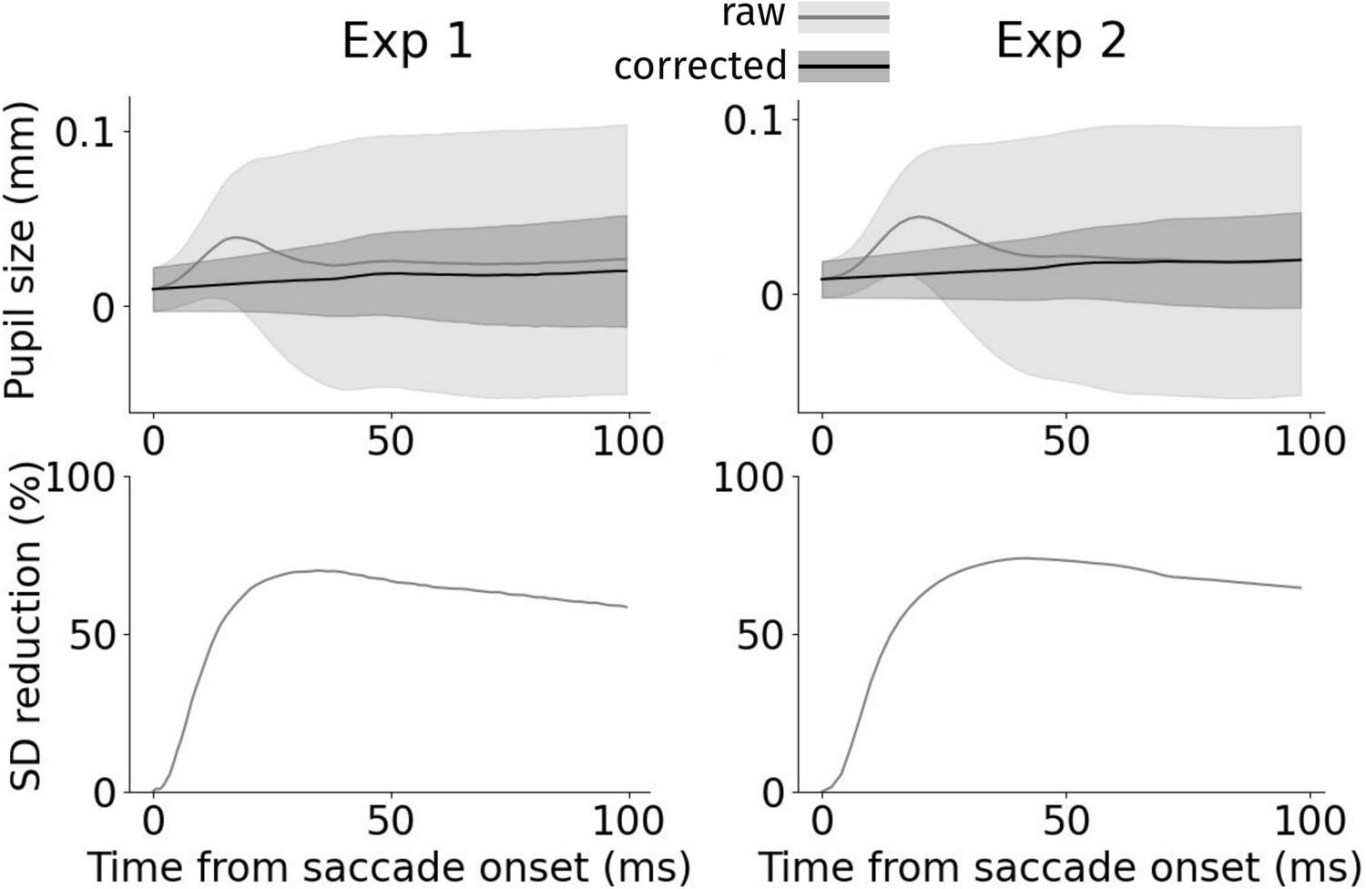
Snip & Stitch substantially reduces pupil size variability after saccade onset. *Top*: Average pupil size change locked to saccade onset (*t* = 0) together with standard deviation across trials with exactly two saccades. *Light gray* denotes uncorrected *pupil* size, *black* denotes corrected pupil size. *Error bands* denote standard deviation. *Bottom*: percentage reduction in standard deviation over time through Snip & Stitch. *Left*: Experiment 1, *Right*: Experiment 2. The average saccade duration was 45.8 ms (*SD*: 7.33 ms) for Experiment 1 and 65.29 ms (*SD*: 8.11 ms) for Experiment 2

The outcome of this correction is shown in Fig. 5b. As expected, the linear correction almost completely removed the systematic build-up of leftover error, yet its standard deviation remained unchanged. From these results, we estimate that with the most elaborate version of Snip  & Stitch, sequences of around ten saccades can be corrected before (accumulated systematic) leftover error introduced by the correction exceeds the error introduced by the PFE in the first place.

### Alternative evaluation measures provide converging evidence for the effectiveness of Snip  & Stitch

**Fig. 7:**
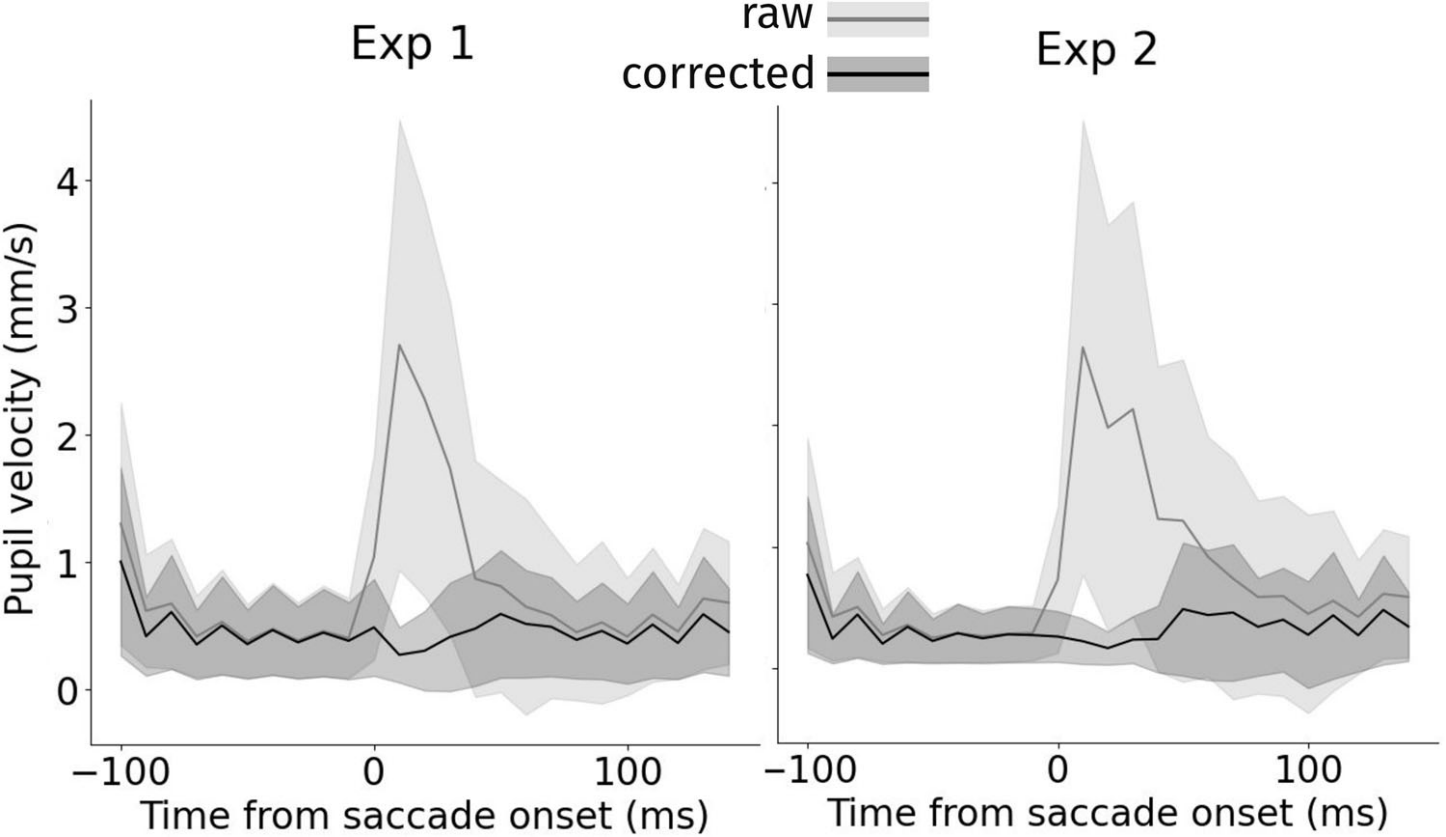
Snip & Stitch corrects for non-plausible pupil size changes during saccade events, thus reducing average pupil size change (velocity). *Light gray* denotes uncorrected data, *black* denotes corrected data. *Error bands* denote standard deviation. *Left*: Experiment 1, *Right*: Experiment 2. The average saccade duration was 45.8 ms (*SD*: 7.33 ms) for Experiment 1 and 65.29 ms (*SD*: 8.11 ms) for Experiment 2

One may worry that not correcting at all would result in a perfect correction as per the corrected versus uncorrected trace comparison in the end of trials. To provide an independent evaluation, we therefore provide an additional test in the following. Specifically, without correction, saccades induce considerable variation in the pupillary signal that merely arises from PFEs rather than physiologically caused variability. A successful correction would thus show in reduced pupillary standard deviation across trials over time, especially from saccade onset. Indeed, we found the standard deviation across trials locked to saccade onset to substantially reduce when Snip  & Stitch + Slope was applied (see Fig. 6). Hereby, not all variability can be considered artifactual in the first place, yet the reduction of the variability comes close to the reductions reported earlier. Additionally, we analyzed average pupil velocity over time (i.e., changes from time point to time point). We expected increased pupil velocity around saccades when the PFE remains uncorrected for. Any successful correction should thus reduce average pupil velocity back to a baseline rate. Indeed, Snip & Stitch was highly successful in reducing pupil velocity around saccades (see Fig. 7).

Together, our results demonstrate that Snip  & Stitch straightforwardly corrects the PFE across different eye trackers, different eccentricities, different saccade directions, and may be effective for sequences of up to ten saccades. To make Snip  & Stitch easy-to-use and accessible, we provide it as a fully open-source Python package.

### Open-source Snip  & Stitch package

We have made Snip  & Stitch openly available via GitHub, see https://github.com/koertstribos/snipandstitch. The package allows to use all Snip  & Stitch functionalities described in this paper on data stored in basic Python objects. Additionally, we provide functions to use all Snip  & Stitch functionalities on data stored in the popular mne toolkit for storing and manipulating eye-tracking and other neurophysiological data in Python (mne.tools). One can install the package via pip (pip install git+https://github.com/koertstribos/snipandstitch) or by copying the files from the GitHub page manually and placing them in their working folder. Working with the Snip  & Stitch package requires minimal information relative to the experimental setup. Users need only provide a list of saccade on- and offsets (we recommend using I2MC; Hessels et al., 2017) in combination with a pupil trace to apply the basic Snip & Stitch algorithm. To utilize the recommended Snip & Stitch + Slope algorithm, users are required to additionally input sampling frequency, as a 100 ms window needs to be defined in sample space.

The package corrects data on a trial-by-trial basis, and comes with the option to perform a correction for systematic build-up over a set of trials, which the user can apply using one function. It should be noted that using this correction assumes that trials start and end with gaze at roughly the same position. Furthermore, we provide functions that apply the corrections described in this paper to example data. We describe how users can implement Snip  & Stitch on their own data in detail on the GitHub page.

## Discussion

The pupil foreshortening error (PFE) restrains the flexibility of pupillometric analysis because researchers need to either ensure constant gaze locations across conditions or gaze-related errors need to be corrected for with complex methods that often require measures unavailable for existing data. To ameliorate this, we here introduced Snip  & Stitch, a straightforward method for correcting for such gaze-based artifacts on pupil size. The simplest version of Snip  & Stitch reduced the PFE by 71%, and our most advanced implementation corrected for approximately 81% of the PFE.

Although extant methods achieve slightly higher performance (e.g., Hayes and Petrov, 2016), our method can be applied, including post hoc, to every dataset containing saccades without the need for complex measurements or cumbersome calibration procedures.

We chose to forego direct comparisons between Snip & Stitch and other methods (i.e., correcting the same data with different techniques) as we did not believe that a fair comparison was possible given the collected data. First, we were unable to directly compare Snip  & Stitch with the Hayes and Petrov (2016) method because we did not have access to the geometric measurements necessary to apply the correction. Furthermore, data from Experiment 2 were collected using the EyeLink 1000 tower mount tracker which uses an intermediary mirror, invalidating a potential geometrically derived PFE correction. Second, we chose not to compare directly with other mapping methods (e.g., Gagl et al., 2011; Brisson et al., 2013) because such methods may ’correct’ true effects of pupil size on gaze location (Jainta et al., 2011; Koevoet et al., 2023) (see Mathôt et al., 2015a, forasimilarpoint).

We made Snip  & Stitch openly available and easy to implement by creating an open-source Python package available at: https://github.com/koertstribos/snipandstitch. The package allows users to apply the methodology described in this paper on pupillometric data in basic Python format, or in format provided by the popular mne toolkit (mne.tools).

We observed a systematic build-up of leftover error when using Snip  & Stitch + slope to correct many saccades in a row. We attribute this build-up to true pupil size dynamics during saccades (e.g., Knapen et al., 2016; Koevoet et al., 2013; Koevoet et al., 2023), which are masked by the Snipping procedure and may not cancel out between forward and return saccades like the PFE. For some observers, this may be a consistent *constriction*, causing a smaller pupil size at the moment observers returned gaze at the screen center. For other observers, in-flight pupillary *dilation* might be masked consistently by our snipping procedure, thus reversing the effect. We also noted that the built-up leftover error exhibited a linear trend per participant. Thus, we implemented a per-saccade correction based on the average observed leftover error per saccade over each experiment and participant to correct for this effect. Built-up leftover error was reduced greatly, while the standard deviation of built-up leftover error remained relatively high. This correction seems to be effective when comparing many trials, but not on a trial-by-trial level. It is important to note that such a correction across trials is less feasible when observers do not move their gaze back to the screen center or when the build-up is potentially less systematic, such as in experiments with more variable gaze target locations and saccade onsets across trials.

We see room for further improvements to Snip  & Stitch. One example is the implementation of a better intrasaccadic pupil size estimation. Snip  & Stitch + Slope assumes that pupil size change during a saccade can be correctly inferred by fitting a linear regression to pupil size just before the saccade and extrapolating this trend. A more complex model might yield better performance. We do not know of any available model that predicts pupil size *during* saccades. Understanding how pupil size changes intrasaccadically may thus improve the correction at hand. Secondly, an improvement to Snip  & Stitch could be made by better correcting the systematic build-up of leftover error that is introduced through repeated applications of Snip  & Stitch in a row. The method described in this paper obtained a per-saccade correction different per participant, but as saccades differ in duration, amplitude, etc., this estimation might work better for some saccades than for others. It is therefore conceivable that different independent variables, such as saccade duration, amplitude, eccentricities, etc., can be observed, and a correct build-up of leftover error will yield better performance, potentially also decreasing the standard deviation in built-up leftover error. Another avenue for improvement may lie in making Snip & Stitch adaptive to pupil size at baseline gaze direction. Possibly, larger pupils necessitate slightly adjusted correction relative to smaller pupils.

As Snip  & Stitch performance depends on a saccade detection algorithm to detect saccade on- and offsets, Snip  & Stitch might be improved by the implementation of a different saccade detection algorithm, or by changing how Snip  & Stitch uses the supplied saccade on-, and offsets to arrive at a PFE estimate. When gaze lands after a saccade, a ’glissade’ might occur, which is a wobbling movement of gaze position at the end of a saccade (Nyström & Holmqvist, 2010; Yamagishi et al., 2020). Moreover, saccade ends exhibit small movement of the pupil relative to the iris (Nyström et al., 2013), meaning that even when an eye has reportedly stopped moving, pupil size estimations might still be affected by the PFE. These complicate the decision of when a saccade ends precisely. A future version of Snip  & Stitch could build on a saccade detection algorithm that separately marks glissades following saccades; this information may help to better estimate post-saccadic pupil size in turn. In theory, Snip  & Stitch does not have to use the full duration of saccades for sufficient correction. For instance, when a saccade ends with a long-lasting glissade or a corrective movement, the effect of the PFE on the pupil at the end of the entire saccade can already be obtained the first time gaze passes or nears the final landing position of the saccade. Implementing such a change will reduce the duration over which pupil size changes are discarded, yielding a more accurate estimate of pupil size after the saccade, as the true pupil size has less time to change in unexpected ways.

Further research may focus on Snip  & Stitch’s applicability across different types of eye trackers. We here demonstrated that Snip  & Stitch effectively reduced the PFE on data from two state-of-the-art eye trackers. This includes the EyeLink Towermount, for which some previous methods could not reduce the PFE due to refractive surfaces used in the eye tracking setup (e.g., Hayes and Petrov, 2016), a problem that should extend to other tower-mounted trackers. It is possible that Snip  & Stitch could offer a solution to the PFE for wearable eye-tracking devices, where they are particularly severe, as the camera recording pupil size is closer to the eye, and therefore affected more by the relative movement of the eye. For wearable eye tracking devices, correcting the PFE has only been shown to be feasible at the population level (Petersch & Dierkes, 2022). In any case, more work is necessary to see how Snip  & Stitch functions when using different eye trackers, including wearables. Ideally, future work will compare the performance of existing PFE corrections/mitigation strategies across trackers, setups, and perhaps tasks and participants. This could also contain scenarios during which the geometry of participants relative to the eye-tracker is variable, i.e., in the absence of chin- and forehead rests.

To conclude, Snip  & Stitch comes with up-, and downsides when compared to alternative PFE corrections, complementing the toolbox of existing PFE correction algorithms. For choosing which method best suits an experiment, we offer some advice on when Snip  & Stitch should and should not be used in the following.

### When (not) to use

Naturally, if participants maintain strict fixation throughout an experiment, gaze affects pupil size only to a very limited extent. In such situations, there is no need to apply Snip & Stitch (or any other PFE correction, for that matter). Similarly, in cases where gaze is shifted, but such shifts are similar or equal across conditions, a correction is unnecessary (e.g., Koevoet et al., 2025; Mathôt et al., 2015b). We note, however, that in such cases, gaze behavior must be closely analyzed to ensure gaze cannot account for potential differences in pupil size between conditions.

In cases where pupil size is affected differently by gaze between conditions, it is important to correct for the PFE to allow for fair comparisons between conditions (e.g., in the case of comparing overt with covert spatial attention, Koevoet et al., 2023). In principle, the Hayes and Petrov correction (2016) is the preferred method, as it has the highest accuracy and does not come with the aforementioned limitations, such as correcting away true cognitive effects. Nevertheless, Snip & Stitch is recommended in (at least) the following scenario’s: if researchers 1) analyze existing datasets for which geometric information about the setup is unavailable, 2) analyze gaze data collected from setups with intermediary mirrors (e.g., EyeLink towermount or in fMRI setups) or 3) if researchers with limited resources or time are not able to obtain material necessary to apply the optimized Hayes and Petrov method (e.g., an artificial eye).

However, Snip & Stitch cannot be applied in all scenarios. We describe important caveats hereunder: One known limitation of Snip & Stitch is that it relies on saccade detection to correct for sudden changes in pupil size caused by eye movements. We therefore recommend researchers to apply state-of-the-art saccade detection algorithms (instead of relying on manufacturers’ saccade-detection), such as I2MC (Hessels et al., 2017) to ensure the best performance of Snip & Stitch. Moreover, if tasks include more gradual shifts in gaze, for example, through smooth pursuit, Snip & Stitch is unable to correct PFE errors.

Furthermore, Snip  & Stitch corrects the PFE, but introduces slight systematic leftover error, decreasing accuracy in reported pupil size over each saccade that is corrected in sequence. Thus, for research and applications in which only few saccades are performed in sequence before participants fixate back at a reference point, Snip  & Stitch substantially reduces the PFE, akin to much more complex state-of-the-art methods. Fortunately, such tasks are omnipresent in (cognitive) psychology and neuroscience. For example, our method should effectively correct for the PFE in highly used and influential tasks, including antisaccade (Coe & Munoz, 2017; Munoz & Everling, 2004), oculomotor capture (Theeuwes et al., 1998), memory-guided saccade (Hikosaka & Wurtz, 1983), and saccade planning/preference tasks (Koevoet et al., 2023, 2024b). Usage of Snip  & Stitch in experiments where more than one saccade is performed before gaze returns to a reference location is possible, but comes with less accurate results (Fig. 5b). We estimate that in datasets wherein participants return to a given reference point (i.e., central fixation) maximum of ten saccades, the PFE can still be reduced using Snip  & Stitch.

Together, the here introduced PFE-correction method complements existing methods, allowing researchers to conduct pupillometric research across a wide range of tasks and situations and reanalyze existing data accordingly.

## Supplementary Information

Below is the link to the electronic supplementary material.

Supplementary file 1 (pdf 426 KB)

## Data Availability

All data can be retrieved from the Open Science Framework: https://osf.io/nu75x.

## Code Availability

All analysis code can be retrieved via OSF: https://osf.io/nu75x. Snip  & Stitch can be accessed via GitHub https://github.com/koertstribos/snipandstitch.

## Declarations

### Open practices statement

This research was not preregistered.
