# Supplementary material for: Snip & Stitch: a simple and accessible correction for the pupil foreshortening error
Source: Behav Res Methods. 2026 Mar 30;58(4):95. doi: 10.3758/s13428-026-02980-8 (PMC13035544; doi:10.3758/s13428-026-02980-8)
Supplement: Supplementary file 1 — (pdf 426 KB) [file 13428_2026_2980_MOESM1_ESM.pdf]

Supplementary Materials to: *Snip & Stitch: A simple and accessible correction for the pupil foreshortening error*

Koert Stribos, Damian Koevoet, Yuqing Cai, Marnix Naber, and Christoph Strauch*

Experimental Psychology, Helmholtz Institute, Utrecht University, The Netherlands

*Correspondence to c.strauch@uu.nl

Absolute Median Error reduction in Snip&Stitch + Slope + Correct

Formulae

Before correction saccades were extended by one sample to ensure that no in-flight samples are used. Thereafter, pupil size at each saccade on- and offset are estimated as median of four neighboring samples. These values are then used to arrive at the basic Δ PFE values:

$$p_{on} = \mathrm{med}(p_{on-1}, p_{on-2}, p_{on-3}, p_{on-4}) \tag{1a}$$

$$p_{off} = \mathrm{med}(p_{off+1}, p_{off+2}, p_{off+3}, p_{off+4}) \tag{1b}$$

$$\Delta PFE = p_{off} - p_{on} \tag{1c}$$

Consequently, pupil sizes after saccade offset are corrected by subtracting ΔPFE, and pupil size during saccade is interpolated linearly between the corrected start and end value:

$$p_{corr}\,(p\,,\,t\,) = \begin{Bmatrix} p\,, & \text{for} \quad t \leq t_{on} \\ p - \Delta PFE\,, & \text{for} \quad t \geq t_{off} \\ p_{on-1} - \frac{\Delta PFE*(t-t_{on})}{(t_{off}-t_{on})}\,, & \text{for} \quad t_{on} > t < t_{off} \end{Bmatrix} \tag{2}$$

The two alternative versions of Snip&Stitch use a modified version of ΔPFE in their correction. For the Snip&Stitch + Slope, an intra-saccadic slope β_{is} is first fit to the first 100 milliseconds before each saccade, and ΔPFE of corresponding saccades is subsequently corrected by multiplying this slope by that saccades' duration.

For the Snip&Stitch + Slope + Correct method, ΔPFE is ultimately corrected with a participant-wide value β_{err}. This value represents per-saccade offset, measured using chains of saccades that start and end at a comparable location (e.g., a central fixation).

$$\Delta PFE_{slope} = \Delta PFE - \beta_{is} * (t_{off} - t_{on}) \quad (3a)$$

$$\Delta PFE_{corr} = \Delta PFE_{slope} - \beta_{err} * 1 \quad (3b)$$

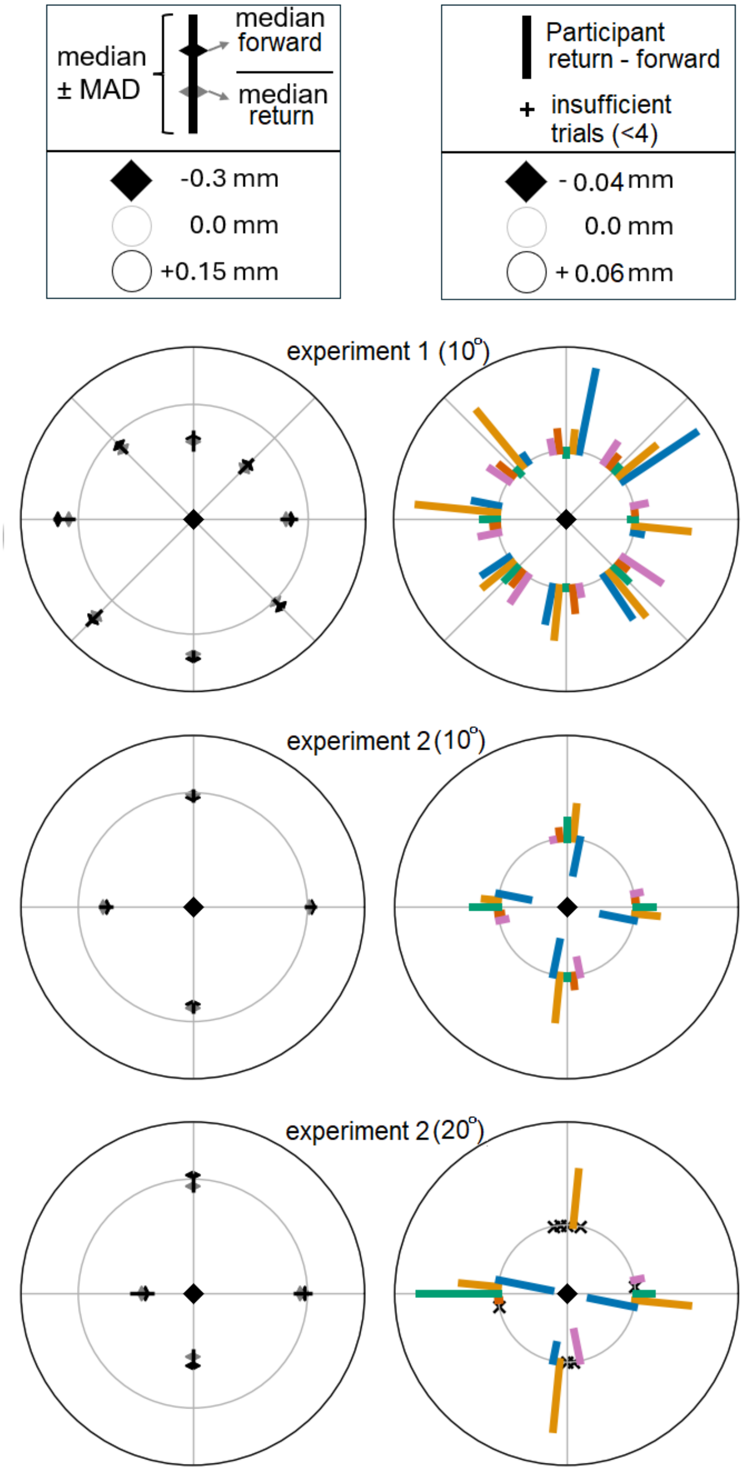

Supplementary Figure 1: This figure illustrates median estimated effect of PFE (see text) over all trials with 2 saccades per direction (left column). For each target, the range of median PFE $\pm$ median absolute deviation is given as black line. Additionally, the median values of only forward (black) and return (grey) saccades are shown. This illustrates an asymmetry, where average PFE is estimated lower from return saccades than from forward saccades. Additionally, (right colum) noted asymmetry estimate per participant x target (right column). Missing values (less than four available trials) are marked with a black cross.

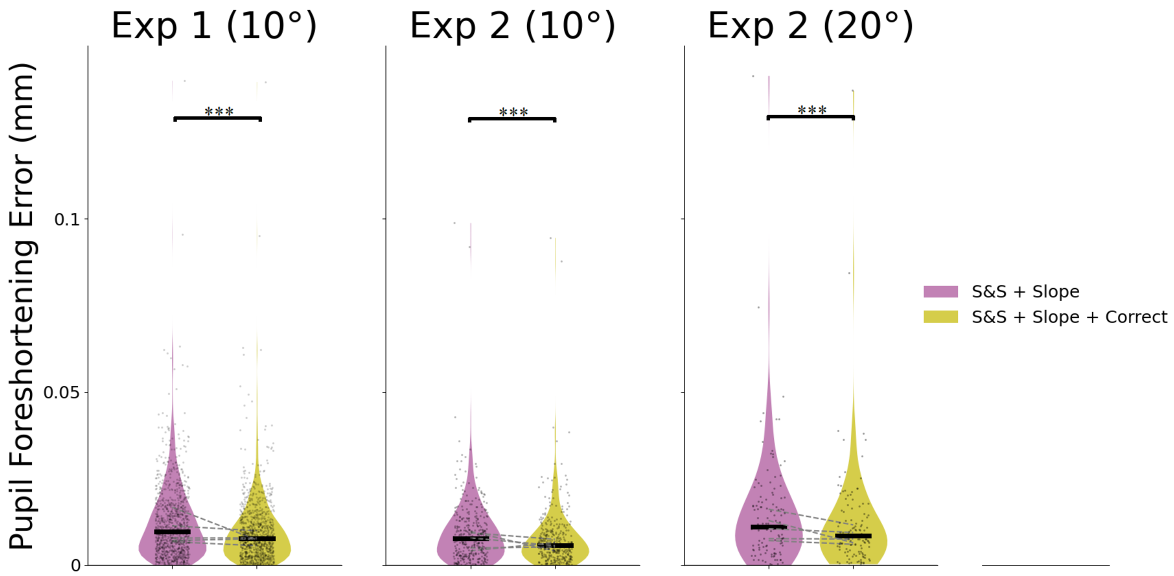

Supplementary Figure 2: This figure illustrates the absolute median error after two saccades for the 'Snip&Stitch + Slope' and the 'Snip&Stitch + Slope + Correct' methods. ***$p < .001$ (one-sided).
